# Гормон роста и фертильность: молекулярные механизмы и опыт адъювантной терапии в репродуктологии

**DOI:** 10.14341/probl13780

**Published:** 2026-09-08

**Authors:** Н. Н. Волеводз, М. В. Воронцова, Д. Ю. Невольников, О. Р. Григорян, С. Ю. Воротникова, Р. К. Михеев

**Affiliations:** Национальный медицинский исследовательский центр эндокринологии им. академика И.И. Дедова; Московский областной научно-исследовательский клинический институт им. М.Ф. Владимирского Россия; Endocrinology Research Centre; Moscow Regional Research and Clinical Institute named after M.F. Vladimirsky Russian Federation; Национальный медицинский исследовательский центр эндокринологии им. академика И.И. Дедова; Московский государственный университет им. М.В. Ломоносова Россия; Endocrinology Research Centre; M. V. Lomonosov Moscow State University Russian Federation; Московский государственный университет им. М.В. Ломоносова Россия; M. V. Lomonosov Moscow State University Russian Federation; Национальный медицинский исследовательский центр эндокринологии им. академика И.И. Дедова Россия; Endocrinology Research Centre Russian Federation

**Keywords:** соматотропный, гормон роста, ИФР-1, вспомогательные репродуктивные технологии, фертильность, бесплодие, беременность, somatotropic, growth hormone, IGF-1, assisted reproductive technologies, fertility, infertility, pregnancy

## Abstract

Гормон роста (ГР) и инсулиноподобный фактор роста-1 (ИФР-1) являются одними из важнейших гормонов не только в регуляции постнатального роста и развития, старения и продолжительности жизни, но и репродуктивной функции у женщин. В клинической практике ГР широко применяется в качестве адъювантного препарата для улучшения различных клинических исходов при использовании вспомогательных репродуктивных технологий (ВРТ) с целью увеличенияя количества получаемых ооцитов/эмбрионов, улучшения их качества, повышения частоты беременностей и успешных родов в общей популяции и среди пациенток с дефицитом ГР. За последние три десятилетия роль ГР, ИФР-1 и их в регуляции женской репродуктивной функции привлекает все большее внимание и становится предметом исследований как в эндокринологии, так и в репродуктологии.

## Abstract (en)

Growth hormone (GH) and insulin-like growth factor-1 (IGF-1) are pivotal hormones regulating not only postnatal growth, development, aging, and longevity but also female reproductive function. In clinical practice, GH is widely utilized as an ad juvant therapy to enhance various clinical outcomes in assisted reproductive technology (ART). It is aimed at increasing the yield and quality of oocytes and embryos, as well as improving pregnancy and live birth rates both in the general population and among patients with GH deficiency. Over the past three decades, the roles of GH and IGF-1 in regulating female repro duction have garnered increasing attention, becoming a major focus of research in both endocrinology and reproductive medicine.

## ВВЕДЕНИЕ

Локус гена гормона роста (ГР) человека (GH) расположен на длинном плече 17-й хромосомы (17q22-24) и представляет собой кластер протяженностью около 50 тысяч пар нуклеотидов. Он включает пять генов: гипофизарный ГР (GH1/GH-N), плацентарный вариант ГР (GH2/GH-V), хорионические соматомаммотропины 1 и 2 (CSH1, CSH2), а также псевдоген хорионического соматомаммотропина (CSH-L) [1].

Согласно современным данным, локально продуцируемый ГР и ассоциированные с ним сигнальные каскады играют ключевую роль в регуляции функции яичников млекопитающих. Так, нокаут рецептора ГР (рГР) (ген GHR) у мышей приводит к выраженному снижению фертильности, что коррелирует с клинической картиной у женщин с дефицитом ГР [2]. В ветеринарной практике применение экзогенного ГР в протоколах индукции овуляции и осеменения достоверно повышает частоту наступления беременности у крупного рогатого скота [3].

Глубокое понимание паттернов экспрессии, молекулярных механизмов и сигнальных путей системы ГР/рГР в яичниках человека критически важно для разработки таргетных диагностических и терапевтических стратегий. В частности, оптимизация схем заместительной терапии ГР может стать перспективным подходом в преодолении бесплодия и коррекции овуляторных нарушений.

## ЭКСПРЕССИЯ ГОРМОНА РОСТА В ГИПОФИЗЕ И ВНЕ ЕГО

Гипофизарный ГР (гГР) экспрессируется, как следует из названия, преимущественно в передней доле гипофиза. Его синтез активируется при связывании специфического белка-активатора с промоторными (цис-активными) последовательностями гена GH1 [4]. Данный транскрипционный фактор, известный как Pit-1 (или POU1F1), относится к семейству POU-доменов и локализуется в ядрах соматотрофов, лактотрофов и тиреотрофов [4]. Pit-1 играет определяющую роль в дифференцировке этих клеточных линий, обеспечивая нормальный морфогенез гипофиза и адекватную секрецию ГР у млекопитающих [5]. Клиническая значимость этого механизма подтверждается тем, что мутации генов Pit-1 и GH1 ассоциированы с тяжелыми формами задержки физического развития у детей [6].

Соматотрофы гипофиза служат основным источником системного ГР: после гипофизэктомии или деструкции железы уровень гормона в сыворотке крови становится практически неопределяемым [7]. Тем не менее экспрессия ГР также зафиксирована в ряде внегипофизарных локаций, включая нервную, иммунную, сердечно-сосудистую и репродуктивную системы, а также костную и мышечную ткани [8]. В данных локусах локально синтезируемый ГР выступает в роли аутокринного или паракринного регулятора, контролируя пролиферацию, дифференцировку и метаболизм клеток. Примечательно, что такая локальная регуляция инициируется в онтогенезе еще до окончательного формирования гипофиза [8].

## ЭКСПРЕССИЯ ГОРМОНА РОСТА В ЯИЧНИКАХ

Транскрипты всех пяти генов кластера гормона роста (GH1, GH2, CSH1, CSH2 и CSHL1) выявляются в яичниках женщин как в репродуктивный период, так и в постменопаузе [9]. мРНК и белковые продукты ГР локализованы в ооцитах, клетках стромы и гранулезы [10]. Участие гГР в регуляции ключевых овариальных процессов, в частности фолликулогенеза, свидетельствует в пользу аутокринного и паракринного механизмов действия гормона [11].

Результаты современных исследований, выполненных с применением иммунофлуорометрического анализа, подтверждают наличие белковых продуктов генов GH1, CSH1 и CSH2 (соответственно, гГР, плацентарный лактоген типа А (hPL-A/ПЛГ А) и плацентарный лактоген типа В (hPL-B/ПЛГ В)) в экстрактах тканей яичников независимо от репродуктивного статуса женщины. Примечательно, что концентрация белка гГР остается стабильной в различные этапы репродуктивного старения, в то время как уровни белков семейства плацентарных лактогенов существенно снижаются в постменопаузе [9].

## РЕЦЕПТОРЫ ГОРМОНА РОСТА В ЯИЧНИКАХ

Исследования на животных моделях (крысы, свиньи, крупный рогатый скот) продемонстрировали экспрессию рецептора гормона роста (рГР) в ооцитах и клетках гранулезы преантральных фолликулов [12]. У человека белок рГР и его мРНК-транскрипты идентифицированы в ооцитах, клетках гранулезы и строме яичников как в антенатальном периоде, так и у взрослых женщин [10]. Кроме того, экспрессия рГР зафиксирована в клетках желтого тела у различных видов млекопитающих, включая человека, что свидетельствует о значимой роли соматотропной системы в регуляции лютеиновой функции [6].

## ВЛИЯНИЕ ГОРМОНА РОСТА НА ЯИЧНИКИ И МАТКУ

ГР способствует повышению фертильности, оказывая прямое воздействие на яичники и матку [13]. Обладая антиоксидантными свойствами, ГР минимизирует повреждение тканей (в том числе после хирургических вмешательств), улучшая общее функциональное состояние яичников. Локально синтезируемый ГР способен связываться с соответствующим рецептором (рГР) в эндоплазматическом ретикулуме. Образующийся комплекс ГР/рГР инициирует рекрутирование и аутофосфорилирование киназы JAK2 на цитоплазматическом домене рецептора [14]. В дальнейшем комплекс рГР/JAK2 индуцирует фосфорилирование белков семейства STAT (STAT5a, STAT5b, STAT1 и STAT3), которые транслоцируются в ядро, модифицируя экспрессию генов и клеточную активность, включая процессы пролиферации [15].

При взаимодействии гонадотропинов (ФСГ и ЛГ) со специфическими рецепторами (рФСГ и рЛГ) активируется сигнальный путь цАМФ/протеинкиназа А, запускающий ключевые фолликулярные процессы: стероидогенез, пролиферацию и дифференцировку клеток [16]. Исследования на культуре клеток гранулезы крыс показали, что ГР потенцирует ФСГ-индуцированную дифференцировку за счет повышения экспрессии рФСГ [17]. Синергичное действие ГР и ФСГ-зависимого цАМФ-сигналинга приводит к увеличению экспрессии рецепторов ЛГ и усилению синтеза прогестерона [18]. У женщин старшего репродуктивного возраста и пациенток с низким овариальным резервом в программах эстракорпорального оплодотворения (ЭКО) включение ГР в протоколы стимуляции сопровождается повышением экспрессии рЛГ, рФСГ и рГР в клетках гранулезы [19]. Таким образом, ГР способен повышать чувствительность клеток гранулезы к гонадотропинам, модулируя стероидогенез и развитие фолликулов [20].

Накопленные данные подтверждают роль ГР как одного из ключевых регуляторов стероидогенеза, фолликулогенеза, созревания ооцитов, частоты овуляции и функциональной активности желтого тела [13]. Несмотря на то, что гонадотропины гипофиза остаются доминирующими регуляторами овариального стероидогенеза, исследования in vitro демонстрируют способность ГР модулировать продукцию эстрадиола и прогестерона [21]. В частности, в клетках гранулезы крупного рогатого скота и лютеинизированных клетках гранулезы человека ГР потенцирует синтез этих стероидных гормонов [13].

Эффекты ГР на стероидогенез имеют межвидовые различия и зависят от гистологической структуры яичника. Так, у свиней ГР стимулирует базальную продукцию прогестерона в желтом теле, не оказывая влияния на развивающиеся фолликулы. При этом ГР усиливает лептин-индуцированный синтез прогестерона фолликулом в периоде перехода в желтое тело. В клетках гранулезы крыс ГР активирует ФСГ-зависимый стероидогенез, не влияя на его базальный уровень. Кроме того, установлено, что ГР способен стимулировать синтез андростерона и других андрогенов в клетках теки крыс вне зависимости от продукции ИФР-1 и цАМФ-сигналинга [6].

Экспериментальные данные на моделях животных подтверждают пролиферативное и антиапоптотическое действие ГР на овариальные фолликулы. Установлено, что введение экзогенного ГР способствует увеличению размеров и количества фолликулов, а также общей массы яичников [11]. На ранних стадиях фолликулогенеза активация примордиальных фолликулов и их последующая трансформация в преантральные структуры протекают независимо от гонадотропинов и регулируются локальными факторами роста по аутокринному и паракринному механизмам [22]. ГР, с большой вероятностью, выступает одним из важных участников этого процесса. Продемонстрировано, что нокаутирование гена рГР (Ghr) у мышей приводит к накоплению примордиальных фолликулов на фоне снижения пула последующих форм с развитием их интенсивной атрезии [23][38]. У животных с дефицитом ГР наблюдается снижение частоты овуляций, имплантаций и численности помомства [23]. При этом терапия экзогенным ГР восстанавливает популяцию развивающихся фолликулов у различных видов млекопитающих с соматотропной недостаточностью [24].

Роль ГР и его сигналинга сохраняется и на поздних этапах развития фолликулов. В частности, нечувствительность тканей к ГР (вследствие дефекта или отсутствия рецептора рГР) ассоциирована с полной остановкой селекции и развития доминантного фолликула, что было продемонстрировано на моделях крупного рогатого скота [25].

Особый интерес представляет влияние ГР на качество ооцитов. Экспериментальные данные подтверждают, что добавление ГР in vitro ускоряет процессы ядерного созревания у различных видов млекопитающих [6]. Показано, что данный эффект реализуется через стимуляцию путем активации экспансии клеток кумулюса (маркер качества ооцита) и супрессию экспрессии коннексина 43 (основного белка щелевых контактов) за счет ингибирования апоптоза и снижения синтеза. Помимо влияния на ядерный аппарат, ГР способствует цитоплазматическому созреванию, так его добавление in vitro значимо улучшает кортикальное распределение органелл в ооцитах лошадей и коров [6].

Регуляторная роль ГР также проявляется в модуляции количества высвобождаемых ооцитов у полиовуляторных видов. У трансгенных мышей с гиперэкспрессией ГР наблюдается увеличение частоты овуляций и численности приплода, в то время как у животных с нокаутом рецептора рГР (мутация GHR-/-, имитирующая синдром Ларона) наблюдается снижение различных параметров фертильности, включая задержку полового созревания, парадоксальное замедленное истощения фолликулярного резерва за счет замедления роста примордиальных фолликулов и уменьшение численности потомства, не связанное с негативным на имплантацию или формирование плаценты [26][40][41]. Примечательно, что данный субфертильный фенотип является частично обратимым при системном введении ИФР-1, что свидетельствует о медиаторной роли ИФР-1 в непосредственной регуляции фолликулогенеза [23].

Кроме того, применение ГР в протоколах суперовуляции у овец позволяет увеличить выход ооцитов [27], а сочетанное использование ГР и ФСГ у данного вида повышает численность пригодных для переноса эмбрионов за счет снижения доли неоплодотворенных яйцеклеток и дегенеративных эмбрионов [28]. Сходный эффект наблюдается и у самок молодых свиней, ежедневное введение ГР повышает частоту овуляций, однако длительная терапия (более 9 дней) приводит к супрессии последующего полового цикла [29].

Поскольку рецепторы к ГР экспрессируются в лютеиновых клетках многих млекопитающих, данный гормон выступает важным регулятором функции желтого тела, оказывая пролиферативное и антиапоптотическое действие [21]. ГР стимулирует пролиферацию лютеинизированных клеток гранулезы человека и ингибирует апоптоз в желтом теле свиней [30]. Напротив, у мышей с нокаутом гена рГР (Ghr) наблюдается повышение интенсивности апоптоза в антральных фолликулах и уменьшение их общего пула [26]. Однако заместительная терапия экзогенным ГР позволяет нормализовать гистологическую картину яичников, а при введении гормона наблюдается активация покоящихся примордиальных фолликулов и восстановление численности антральных фолликулов [43].

Поддерживающее влияние ГР на функциональную активность желтого тела может быть опосредовано стимуляцией синтеза прогестерона, который сам по себе является мощным антиапоптотическим фактором [31]. Кроме того, на ранних стадиях формирования желтого тела у крупного рогатого скота ГР in vitro, совместно с другими регуляторными пептидами, индуцирует секрецию простагландина F2α, что на данном этапе способствует продукции прогестерона и поддержанию жизнеспособности лютеиновой ткани [32].

## СИНЕРГИСТ ГР — ИФР-1

Накопленные данные свидетельствуют о сочетанном действии ГР и ИФР-1, которые способствуют дифференцировке клеток теки и гранулезы в лютеиновые клетки [33]. Молекулярный механизм реализации эффектов гГР включает его взаимодействие с рГР, что инициирует рекрутирование и фосфорилирование киназы JAK2 на цитоплазматическом домене рецептора [15]. Активированный комплекс рГР/JAK2 обеспечивает фосфорилирование транскрипционного фактора STAT5b, который индуцирует экспрессию ИФР-1 в гепатоцитах [15][16]. Аналогичный внутриклеточный сигнальный путь ГР/рГР ответственен за локальную продукцию ИФР-1 в клетках гранулезы (продемонстрировано на моделях крыс) [34]. Несмотря на разнообразие нижележащих каскадов ФСГ и ЛГ, современные исследования подтверждают наличие выраженного синергизма между путями ФСГ/ЛГ и ГР/ИФР-1 [33].

Результаты исследований на животных моделях и в клинической практике указывают на то, что наряду с ГР ИФР-1 также выступает регулятором стероидогенеза, клеточной пролиферации и фолликулогенеза [33]. Установлено, что концентрации ГР и ИФР-1 в фолликулярной жидкости достоверно выше в фолликулах, содержащих ооциты с нормальной морфологией, что подтверждает корреляцию уровней данных факторов с качеством женских гамет [35]. ИФР-1 также способствует созреванию ооцитов in vitro, так добавление ИФР-1 в культуральную среду незрелых ооцитов повышает процент созревания и улучшает морфофункциональные их характеристики [36]. Таргетная делеция гена Igf1 у мышей приводит к развитию фенотипа карликовости и бесплодия и характеризуется глубоким нарушением овуляции (как спонтанной, так и индуцированной гонадотропинами), накоплением пула примордиальных и первичных фолликулов на фоне отсутствия антральных [42].

## ГОРМОН РОСТА И КАЧЕСТВО ООЦИТОВ ЧЕЛОВЕКА

При проведении программ ЭКО от 10 до 60% полученных ооцитов имеют различные морфологические аномалии. К ним относятся нарушение формы гаметы, наличие диффузных цитоплазматических вакуолей, гранулярность ооплазмы, рефрактерные тельца, расширение перивителлинового пространства, присутствие детрита, а также увеличение объема или фрагментация первого полярного тельца [44]. Эти дефекты могут быть обусловлены как внутренними факторами (возраст пациентки, генетические нарушения), так и внешними воздействиями (особенности питания, протоколы индукции овуляции, условия культивирования).

Установлено, что уровни ГР и ИФР-1 в сыворотке крови положительно коррелируют с морфофункциональными характеристиками фолликулов, зрелостью ооцитов и концентрацией стероидных гормонов [45]. Анализ фолликулярной жидкости (ФЖ) пациенток в циклах ЭКО выявил прямую связь между содержанием ГР и частотой получения ооцитов, давших начало жизнеспособным эмбрионам для переноса [46]. Сходным образом концентрация ИФР-1 в ФЖ положительно коррелирует с количеством зрелых ооцитов [45]. Результаты проспективных пилотных исследований подтверждают, что уровни ГР и ИФР-1 в фолликулярной жидкости достоверно выше в группах с нормальной морфологией ооцитов (соответственно, и с большей частотой оплодотворения) по сравнению с группами, где более 50% гамет имеют аномалии [47].

Относительно качества эмбрионов данные литературы остаются дискуссионными: в ряде работ высокие концентрации ГР в ФЖ ассоциировались с повышенной скоростью дробления, оптимальной морфологией и высоким имплантационным потенциалом эмбрионов, однако в других исследованиях статистически значимой связи не выявлено. Тем не менее рандомизированные контролируемые исследования с применением протоколов на основе агонистов ГнРГ демонстрируют, что адъювантная терапия ГР увеличивает общее число полученных и зрелых ооцитов, частоту оплодотворения, а также количество перенесенных и криоконсервированных эмбрионов [48]. Показано, что включение ГР в протоколы стимуляции потенцирует ответ яичников на гонадотропины [49]. Кроме того, добавление ИФР-1 в среду для созревания ооцитов in vitro способствует увеличению доли зрелых гамет и снижению частоты их морфологических дефектов [36].

## КЛИНИЧЕСКОЕ ПРИМЕНЕНИЕ ГОРМОНА РОСТА ПРИ ЖЕНСКОМ БЕСПЛОДИИ

## Результаты у женщин с недостаточным ответом яичников на лечение

Клинические данные подтверждают, что концентрация ИФР-1 в ФЖ положительно коррелирует с числом растущих фолликулов и отрицательно — с суммарной дозой и продолжительностью гонадотропной стимуляции яичников [50], а высокие уровни ГР в ФЖ ассоциированы с более эффективным оплодотворением, качеством эмбриогенеза, а также повышенной частотой имплантации и наступления беременности [46]. Несмотря на это, в ряде исследований не было выявлено значимого улучшения клинических исходов при включении ГР в общие протоколы ЭКО [6]. В связи с этим дальнейший научный поиск сосредоточился на применении ГР исключительно у пациенток с «бедным» овариальным ответом. Хотя экспериментальные модели демонстрируют явные преимущества ГР в регуляции фолликулогенеза, клиническая значимость его адъювантного использования в программах ВРТ для определенных групп пациентов остается предметом дискуссий из-за существенной гетерогенности критериев включения в существующих исследованиях [6]. Эффективность применения ГР у пациенток с «бедным» ответом была проанализирована в нескольких метаанализах (табл. 1).

**Table:** Таблица 1. Сводная таблица метаанализов по эффективности ГР в программах ВРТ

Год публикации метаанализа	Статус пациенток	Материалы исследования (количество РКИ и пациенток)	Результат
Duffy J.M. и соавт., 2021	«Бедный» ответ	15 РКИ/1290 чел.	Применение гормона роста у пациенток с «бедным» ответом яичников приводит к достоверному увеличению частоты живорождения и клинической беременности
Zhang Y. и соавт., 2020 [55].	«Бедный» ответ	46 РКИ (разные адъюванты)	ГР показал высокую эффективность по сравнению с другими адъювантными препаратами в увеличении количества полученных ооцитов и эмбрионов у пациенток с «бедным» ответом яичников
Cozzolino M. и соавт., 2020	«Бедный» ответ	12 РКИ/1139 чел.	Подтверждено значимое повышение частоты живорождения и клинической беременности у пациенток с «бедным» ответом яичников при использовании ГР
Li X.L. и соавт., 2017 [53]	«Бедный» ответ	11 РКИ/1083 чел.	Установлено значимое увеличение общего числа полученных ооцитов.Значительное повышение частоты клинической беременности и живорождения
Yu X. и соавт., 2015 [54].	«Бедный» ответ	12 РКИ/984 чел.	Включение ГР в протоколы стимуляции способствует повышению сывороточного уровня эстрадиола (в день введения триггера ХГЧ), увеличению числа ооцитов на стадии MII, количества эмбрионов с двумя пронуклеусами и общего числа эмбрионов для переноса
Jeve Y.B. и соавт., 2014	«Бедный» ответ	10 РКИ/724 чел.	Увеличение вероятности живорождения более чем в 2 раза у пациенток со сниженным овариальным резервом
Duffy J.M. и соавт., 2010	«Бедный» ответ	10 РКИ/448 чел.	Для пациенток с «бедным» ответом зафиксирован рост частоты наступления беременности и показателя живорождения.При нормальном ответе отмечено полное отсутствие эффекта от добавления ГР
Kyrou D. и соавт., 2009 [52]	«Бедный» ответ	22 РКИ/-	ГР признан эффективным дополнением к стимуляции при сравнении нескольких стратегий адъювантной терапии
Kolibianakis E.M. и соавт., 2009 [51]	«Бедный» ответ	6 РКИ/169 чел.	Применение ГР привносит статистически значимое преимущество по частоте живорождения у женщин со сниженным ответом яичников
Harper K.M. и соавт., 2003 [56]	Смешанная группа	6 РКИ/141 чел.	Применение ГР значительно сокращает общую дозировку гонадотропинов, необходимых для стимуляции яичников. Отсутствие пользы для пациентов с нормальным овариальным ответом, тенденция к пользе при «бедном» ответе

Несмотря на полученные данные, влияние адъювантной терапии ГР на частоту живорождения у данной категории пациенток остается неопределенным ввиду высокой вариабельности дозировок и режимов введения препарата в проанализированных исследованиях. Для окончательного определения роли ГР в программах ЭКО у пациенток со сниженным овариальным резервом необходимо проведение дальнейших крупномасштабных высококачественных РКИ.

## Результаты у женщин с низким качеством ооцитов или нарушением развития эмбрионов

Низкое качество ооцитов и последующее нарушение эмбриогенеза являются основными причинами неудач программ ЭКО или переносов эмбрионов. Эта проблема актуальна и для пациенток с нормальным овариальным ответом, так как существует тесная взаимосвязь между морфофункциональными характеристиками ооцитов/эмбрионов и частотой наступления беременности [57]. Исследования показывают, что ГР способен улучшать митохондриальную функцию, а также ядерное и цитоплазматическое созревание ооцитов у пациенток старшего репродуктивного возраста и женщин с «бедным» ответом, что определяет потенциал включения ГР в протоколы стимуляции с целью увеличенияя числа зрелых ооцитов и повышения качества полученных эмбрионов [51].

В исследовании Li et al., включавшем 158 пациенток с неудачными попытками ЭКО в анамнезе из-за отсутствия эмбрионов высокого качества, применение ГР в дозе 3 МЕ/сутки привело к достоверному увеличению количества полученных ооцитов и дробящихся эмбрионов. Это, в свою очередь, обеспечило более высокие показатели имплантации, клинической беременности и живорождения. Примечательно, что клетки кумулюса в группе ГР характеризовались более высоким числом копий митохондриальной ДНК по сравнению с контролем, что указывает на потенциальное положительное влияние ГР на энергетический статус ооцитов и их потенциал к дальнейшему развитию [58]. Систематический обзор Duffy et al. подтверждает данные выводы, указывая, что адъювантная терапия ГР потенцирует ответ яичников на стимуляцию, увеличивает выход ооцитов и эмбрионов, способствуя росту частоты наступления беременности и живорождения [49].

## БЕЗОПАСНОСТЬ И ПОТЕНЦИАЛЬНЫЕ РИСКИ ПРИМЕНЕНИЯ ГР В ПРОТОКОЛАХ ВРТ

В настоящее время краткосрочное применение ГР в программах ВРТ характеризуются благоприятным профилем безопасности и не вызывает серьезных опасений в клинической практике. Тем не менее использование ГР потенциально сопряжено с рисками при активных онкологических процессах и ряде метаболических нарушений, таких как сахарный диабет [59]. Установлено, что применение соматотропина может индуцировать значимые метаболические изменения, включая развитие инсулинорезистентности, нарушение толерантности к глюкозе, дислипидемию и активацию ренин-ангиотензиновой системы [59].

В то же время систематический обзор van Bunderen et al. продемонстрировал, что длительная заместительная терапия ГР оказывает протективное действие, снижая риск переломов, сердечно-сосудистых осложнений и инсульта, не повышая при этом риск развития злокачественных новообразований [60]. Несмотря на имеющиеся данные о безопасности, клиницистам следует учитывать потенциальные побочные эффекты и проводить всестороннюю индивидуальную оценку состояния пациентки при выборе дозировки и схемы применения ГР в циклах ВРТ.

## ЗАКЛЮЧЕНИЕ

Адъювантная терапия ГР применяется в программах ВРТ на протяжении трех десятилетий. Помимо секреции в передней доле гипофиза, ГР и его функциональный рецептор экспрессируются непосредственно в тканях женской репродуктивной системы, включая яичники и матку. Данные экспериментальных и клинических исследований свидетельствуют о том, что ГР и ИФР-1 играют фундаментальную роль в регуляции ключевых овариальных процессов: активации примордиальных фолликулов, фолликулогенезе, стероидогенезе, созревании ооцитов и обеспечении условий для имплантации.

Концентрации ГР и ИФР-1 в фолликулярной жидкости положительно коррелируют с морфофункциональными характеристиками ооцитов, качеством эмбрионов и скоростью их дробления. Клиническая практика подтверждает, что включение ГР в протоколы стимуляции ассоциировано с улучшением репродуктивных исходов: увеличением числа зрелых ооцитов, повышением частоты оплодотворения, имплантации, наступления клинической беременности и живорождения. Наиболее выраженный терапевтический эффект наблюдается в группе пациенток с «бедным» овариальным ответом. Однако существующая гетерогенность данных, обусловленная методологическими различиями и ограниченным объемом выборок в ряде работ, препятствует формированию единых клинических рекомендаций по оптимальному режиму и дозированию ГР. Для окончательной верификации эффективности ГР в программах ВРТ необходимо проведение масштабных рандомизированных клинических исследований с жестким методологическим контролем.

## ДОПОЛНИТЕЛЬНАЯ ИНФОРМАЦИЯ

Источники финансирования. Статья подготовлена в рамках выполнения государственного задания Минздрава России №НИОКТР 126022417900-6.

Конфликт интересов. Авторы декларируют отсутствие явных и потенциальных конфликтов интересов, связанных с содержанием настоящей статьи.

Участие авторов. Все авторы одобрили финальную версию статьи перед публикацией, выразили согласие нести ответственность за все аспекты работы, подразумевающую надлежащее изучение и решение вопросов, связанных с точностью или добросовестностью любой части работы.

## References

[cit1] Harper ME, Barrera-Saldaña HA, Saunders GF. Chromosomal localization of the human placental lactogen-growth hormone gene cluster to 17q22-24. Am J Hum Genet. 1982;34(2):227-234 PMC1685277 7072716

[cit2] Bartke Andrzej, Sun Liou Y., Longo Valter (2013). Somatotropic Signaling: Trade-Offs Between Growth, Reproductive Development, and Longevity. Physiological Reviews.

[cit3] Starbuck Melanie J., Inskeep E. Keith, Dailey Robert A. (2005). Effect of a single growth hormone (rbST) treatment at breeding on conception rates and pregnancy retention in dairy and beef cattle. Animal Reproduction Science.

[cit4] Parks John S., Abdul-Latif Hussein, Kinoshita Ei-ichi, Meacham Lillian R., Pfaffle Roland W., Brown Milton R. (2008). Genetics of Growth Hormone Gene Expression. Hormone Research.

[cit5] Szeto D P, Ryan A K, O'Connell S M, Rosenfeld M G (2002). P-OTX: a PIT-1-interacting homeodomain factor expressed during anterior pituitary gland development.. Proceedings of the National Academy of Sciences.

[cit6] Chang Chia-Wei, Sung Yu-Wen, Hsueh Ya-Wen, Chen Yi-Yan, Ho Ming, Hsu Hsi-Chen, Yang Tung-Chuan, Lin Wu-Chou, Chang Hsun-Ming (2022). Growth hormone in fertility and infertility: Mechanisms of action and clinical applications. Frontiers in Endocrinology.

[cit7] KARIN MICHAEL, THEILL LARS, CASTRILLO JOSE-LUIS, MCCORMICK ALISON, BRADY HELEN (2013). Tissue-Specific Expression of the Growth Hormone Gene and Its Control by Growth Hormone Factor-1. Proceedings of the 1989 Laurentian Hormone Conference.

[cit8] Harvey Steve, Baudet Marie-Laure (2014). Extrapituitary growth hormone and growth?. General and Comparative Endocrinology.

[cit9] Schwarzler P. (2004). Selective Growth Hormone/Placental Lactogen Gene Transcription and Hormone Production in Pre- and Postmenopausal Human Ovaries. Journal of Clinical Endocrinology & Metabolism.

[cit10] Abir Ronit, Garor Roni, Felz Carmela, Nitke Shmuel, Krissi Haim, Fisch Benjamin (2007). Growth hormone and its receptor in human ovaries from fetuses and adults. Fertility and Sterility.

[cit11] Silva J.R.V., Figueiredo J.R., van den Hurk R. (2009). Involvement of growth hormone (GH) and insulin-like growth factor (IGF) system in ovarian folliculogenesis. Theriogenology.

[cit12] Slater Michael, Cooper M., Murphy C.R. (2006). Human growth hormone and interleukin-6 are upregulated in endometriosis and endometrioid adenocarcinoma. Acta Histochemica.

[cit13] Hull Kerry L., Harvey Steve (2014). Growth Hormone and Reproduction: A Review of Endocrine and Autocrine/Paracrine Interactions. International Journal of Endocrinology.

[cit14] Yigiter Murat, Halici Zekai, Odabasoglu Fehmi, Keles Osman Nuri, Atalay Fadime, Unal Bunyami, Salman Ahmet Bedii (2011). Growth hormone reduces tissue damage in rat ovaries subjected to torsion and detorsion: biochemical and histopathologic evaluation. European Journal of Obstetrics & Gynecology and Reproductive Biology.

[cit15] Rotwein Peter (2012). Mapping the growth hormone–Stat5b–IGF-I transcriptional circuit. Trends in Endocrinology & Metabolism.

[cit16] Orisaka Makoto, Miyazaki Yumiko, Shirafuji Aya, Tamamura Chiyo, Tsuyoshi Hideaki, Tsang Benjamin K., Yoshida Yoshio (2021). The role of pituitary gonadotropins and intraovarian regulators in follicle development: A mini‐review. Reproductive Medicine and Biology.

[cit17] JIA XIAO-CHI, KALMIJN JELGER, HSUEH AARON J. W. (2009). Growth Hormone Enhances Follicle-Stimulating Hormone-Induced Differentiation of Cultured Rat Granulosa Cells*. Endocrinology.

[cit18] Nimrod A. (2002). The induction of ovarian LH‐receptors by FSH is mediated by cyclic AMP. FEBS Letters.

[cit19] Regan Sheena L.P., Knight Phil G., Yovich John L., Arfuso Frank, Dharmarajan Arun (2018). Growth hormone during in vitro fertilization in older women modulates the density of receptors in granulosa cells, with improved pregnancy outcomes. Fertility and Sterility.

[cit20] Weall B M, Al-Samerria S, Conceicao J, Yovich J L, Almahbobi G (2014). A direct action for GH in improvement of oocyte quality in poor-responder patients. Reproduction.

[cit21] Sirotkin A.V. (2004). Control of reproductive processes by growth hormone: Extra- and intracellular mechanisms. The Veterinary Journal.

[cit22] Kezele Phillip (2007). Cell-cell interactions in primordial follicle assembly and development. Frontiers in Bioscience.

[cit23] Slot Karin A, Kastelijn Jan, Bachelot Anne, Kelly Paul A, Binart Nadine, Teerds Katja J (2006). Reduced recruitment and survival of primordial and growing follicles in GH receptor-deficient mice. Reproduction.

[cit24] Sá Filho M.F., Carvalho N.A.T., Gimenes L.U., Torres-Júnior J.R., Nasser L.F.T., Tonhati H., Garcia J.M., Gasparrini B., Zicarelli L., Baruselli P.S. (2008). Effect of recombinant bovine somatotropin (bST) on follicular population and on in vitro buffalo embryo production. Animal Reproduction Science.

[cit25] Chase C C, Kirby C J, Hammond A C, Olson T A, Lucy M C (2016). Patterns of ovarian growth and development in cattle with a growth hormone receptor deficiency.. Journal of Animal Science.

[cit26] Zaczek Denise, Hammond James, Suen Lii, Wandji Serge, Service Darlene, Bartke Andrzej, Chandrashekar Varadaraj, Coschigano Karen, Kopchick John (2005). Impact of Growth Hormone Resistance on Female Reproductive Function: New Insights from Growth Hormone Receptor Knockout Mice1. Biology of Reproduction.

[cit27] Gonzalez-Añover Pedro, Encinas Teresa, Garcia-Garcia Rosa M., Veiga-Lopez Almudena, Cocero Maria J., McNeilly Alan S., Gonzalez-Bulnes Antonio (2005). Ovarian response in sheep superovulated after pretreatment with growth hormone and GnRH antagonists is weakened by failures in oocyte maturation. Zygote.

[cit28] Folch J., Ramón J.P., Cocero M.J., Alabart J.L., Beckers J.F. (2002). Exogenous growth hormone improves the number of transferable embryos in superovulated ewes. Theriogenology.

[cit29] Andres C. J., Green M. L., Clapper J. A., Cline T. R., Diekman M. A. (2016). Influence of daily injections of porcine somatotropin on growth, puberty, and reproduction in gilts2. Journal of Animal Science.

[cit30] GREGORASZCZUK Ewa Lucja, PTAK Anna (2006). In Vitro Effect of Leptin on Growth Hormone (GH)- and Insulin-Like Growth Factor-I (IGF-I)-stimulated Progesterone Secretion and Apoptosis in Developing and Mature Corpora Lutea of Pig Ovaries. Journal of Reproduction and Development.

[cit31] Berisha B., Schams D. (2005). Ovarian function in ruminants. Domestic Animal Endocrinology.

[cit32] Kobayashi Shuichi, Miyamoto Akio, Berisha Bajram, Schams Dieter (2002). Growth hormone, but not luteinizing hormone, acts with luteal peptides on prostaglandin F2α and progesterone secretion by bovine corpora lutea in vitro☆. Prostaglandins & Other Lipid Mediators.

[cit33] Ipsa Emina, Cruzat Vinicius F., Kagize Jackob N., Yovich John L., Keane Kevin N. (2019). Growth Hormone and Insulin-Like Growth Factor Action in Reproductive Tissues. Frontiers in Endocrinology.

[cit34] Dehkhoda Farhad, Lee Christine M. M., Medina Johan, Brooks Andrew J. (2018). The Growth Hormone Receptor: Mechanism of Receptor Activation, Cell Signaling, and Physiological Aspects. Frontiers in Endocrinology.

[cit35] Velazquez M.A., Hadeler K.-G., Herrmann D., Kues W.A., Rémy B., Beckers J.-F., Niemann H. (2012). In vivo oocyte IGF-1 priming increases inner cell mass proliferation of in vitro-formed bovine blastocysts. Theriogenology.

[cit36] Yu Y., Yan J., Li M., Yan L., Zhao Y., Lian Y., Li R., Liu P., Qiao J. (2012). Effects of combined epidermal growth factor, brain-derived neurotrophic factor and insulin-like growth factor-1 on human oocyte maturation and early fertilized and cloned embryo development. Human Reproduction.

[cit37] Brown-Borg Holly M., Borg Kurt E., Meliska Charles J., Bartke Andrzej (2003). Dwarf mice and the ageing process. Nature.

[cit38] Zhou Yihua, Xu Bixiong C., Maheshwari Hiralal G., He Li, Reed Michael, Lozykowski Maria, Okada Shigeru, Cataldo Lori, Coschigamo Karen, Wagner Thomas E., Baumann Gerhard, Kopchick John J. (2002). A mammalian model for Laron syndrome produced by targeted disruption of the mouse growth hormone receptor/binding protein gene (the Laron mouse). Proceedings of the National Academy of Sciences.

[cit39] Oliveira Joselina L. M., Aguiar-Oliveira Manuel H., D’Oliveira Argemiro, Pereira Rossana M. C., Oliveira Carla R. P., Farias Catarine T., Barreto-Filho José A., Anjos-Andrade Fernando D., Marques-Santos Celi, Nascimento-Junior Adão C., Alves Érica O., Oliveira Francielle T., Campos Viviane C., Ximenes Roberto, Blackford Amanda, Parmigiani Giovanni, Salvatori Roberto (2007). Congenital Growth Hormone (GH) Deficiency and Atherosclerosis: Effects of GH Replacement in GH-Naive Adults. The Journal of Clinical Endocrinology & Metabolism.

[cit40] List Edward O., Sackmann-Sala Lucila, Berryman Darlene E., Funk Kevin, Kelder Bruce, Gosney Elahu S., Okada Shigeru, Ding Juan, Cruz-Topete Diana, Kopchick John J. (2011). Endocrine Parameters and Phenotypes of the Growth Hormone Receptor Gene Disrupted (GHR−/−) Mouse. Endocrine Reviews.

[cit41] Dosouto Carlos, Calaf Joaquim, Polo Ana, Haahr Thor, Humaidan Peter (2019). Growth Hormone and Reproduction: Lessons Learned From Animal Models and Clinical Trials. Frontiers in Endocrinology.

[cit42] Baker J, Hardy M P, Zhou J, Bondy C, Lupu F, Bellvé A R, Efstratiadis A (2014). Effects of an Igf1 gene null mutation on mouse reproduction.. Molecular Endocrinology.

[cit43] Saccon Tatiana D., Moreira Fabiana, Cruz Luis A., Mondadori Rafael G., Fang Yimin, Barros Carlos C., Spinel L., Bartke A., Masternak Michal M., Schneider A. (2016). Ovarian aging and the activation of the primordial follicle reserve in the long-lived Ames dwarf and the short-lived bGH transgenic mice. Molecular and Cellular Endocrinology.

[cit44] Sousa Mário, Cunha Mariana, Silva Joaquina, Oliveira Elsa, Pinho Maria João, Almeida Carolina, Sá Rosália, da Silva José Teixeira, Oliveira Cristiano, Barros Alberto (2016). Ultrastructural and cytogenetic analyses of mature human oocyte dysmorphisms with respect to clinical outcomes. Journal of Assisted Reproduction and Genetics.

[cit45] Pellegrini S., Fuzzi B., Pratesi S., Mannelli M., Criscuoli L., Messeri G., Forti G. (2012). Physiology: In-vivo studies on ovarian insulin-like growth factor I concentrations in human preovulatory follicles and human ovarian circulation. Human Reproduction.

[cit46] Mendoza C. (2002). Follicular fluid markers of oocyte developmental potential. Human Reproduction.

[cit47] Scheffler Florence, Vandecandelaere Albane, Soyez Marion, Bosquet Dorian, Lefranc Elodie, Copin Henri, Devaux Aviva, Benkhalifa Moncef, Cabry Rosalie, Desailloud Rachel (2021). Follicular GH and IGF1 Levels Are Associated With Oocyte Cohort Quality: A Pilot Study. Frontiers in Endocrinology.

[cit48] Dakhly Dina M.R., Bassiouny Yasmin A., Bayoumi Yomna A., Hassan Mohamed A., Gouda Hisham M., Hassan Ayman A. (2018). The addition of growth hormone adjuvant therapy to the long down regulation protocol in poor responders undergoing in vitro fertilization: Randomized control trial. European Journal of Obstetrics & Gynecology and Reproductive Biology.

[cit49] Duffy James MN, Ahmad Gaity, Mohiyiddeen Lamiya, Nardo Luciano G, Watson Andrew (2010). Growth hormone for in vitro fertilization. Cochrane Database of Systematic Reviews.

[cit50] Oosterhuis G. J. E., Vermes I., Lambalk C. B., Michgelsen H. W. B., Schoemaker J. (2002). Insulin-like growth factor (IGF)-I and IGF binding protein-3 concentrations in fluid from human stimulated follicles. Human Reproduction.

[cit51] Kolibianakis E.M., Venetis C.A., Diedrich K., Tarlatzis B.C., Griesinger G. (2009). Addition of growth hormone to gonadotrophins in ovarian stimulation of poor responders treated by in-vitro fertilization: a systematic review and meta-analysis. Human Reproduction Update.

[cit52] Kyrou Dimitra, Kolibianakis Efstratios M., Venetis Christos A., Papanikolaou Evangelos G., Bontis John, Tarlatzis Basil C. (2008). How to improve the probability of pregnancy in poor responders undergoing in vitro fertilization: a systematic review and meta-analysis. Fertility and Sterility.

[cit53] Li Xue-Li, Wang Li, Lv Fang, Huang Xia-Man, Wang Li-Ping, Pan Yu, Zhang Xiao-Mei (2017). The influence of different growth hormone addition protocols to poor ovarian responders on clinical outcomes in controlled ovary stimulation cycles. Medicine.

[cit54] Yu X, Ruan J, He LP, et al. Efficacy of growth hormone supplementation with gonadotrophins in vitro fertilization for poor ovarian responders: an updated meta-analysis. Int J Clin Exp Med. 2015;8(4):4954-4967 PMC4483949 26131068

[cit55] Zhang Yu, Zhang Chao, Shu Jing, Guo Jing, Chang Hsun-Ming, Leung Peter C K, Sheng Jian-Zhong, Huang Hefeng (2019). Adjuvant treatment strategies in ovarian stimulation for poor responders undergoing IVF: a systematic review and network meta-analysis. Human Reproduction Update.

[cit56] Harper Katie, Proctor Michelle, Hughes Edward, Duffy James M N (2003). Growth hormone for in vitro fertilization. Cochrane Database of Systematic Reviews.

[cit57] Zhu Jinliang, Lian Ying, Li Ming, Chen Lixue, Liu Ping, Qiao Jie (2014). Does IVF cleavage stage embryo quality affect pregnancy complications and neonatal outcomes in singleton gestations after double embryo transfers?. Journal of Assisted Reproduction and Genetics.

[cit58] Li Jingyu, Chen Qiaoli, Wang Jiang, Huang Guoning, Ye Hong (2020). Does growth hormone supplementation improve oocyte competence and IVF outcomes in patients with poor embryonic development? A randomized controlled trial. BMC Pregnancy and Childbirth.

[cit59] Molitch Mark E., Clemmons David R., Malozowski Saul, Merriam George R., Vance Mary Lee (2011). Evaluation and Treatment of Adult Growth Hormone Deficiency: An Endocrine Society Clinical Practice Guideline. The Journal of Clinical Endocrinology & Metabolism.

[cit60] van Bunderen Christa C., van Varsseveld Nadège C., Erfurth Eva Marie, Ket Johannes C.F., Drent Madeleine L. (2014). Efficacy and safety of growth hormone treatment in adults with growth hormone deficiency: a systematic review of studies on morbidity. Clinical Endocrinology.

